# Myths about Oral Health and Associated Factors in Pregnant Women in a Public Hospital in Peru

**DOI:** 10.3290/j.ohpd.c_1845

**Published:** 2025-02-20

**Authors:** Marolyn Leila Vera-Carpio, Kilder Maynor Carranza-Samanez, Julissa Amparo Dulanto-Vargas

**Affiliations:** a Marolyn Leila Vera-Carpio Graduate Student, School of Dentistry, Universidad Científica del Sur, Lima, Perú. Idea, study design, questionnaire design, acquisition of data, wrote and approved the manuscript.; b Kilder Maynor Carranza-Samanez Research Associate, Research Group in Dental Sciences, School of Dentistry, Universidad Científica del Sur, Lima, Perú. Idea, study design, questionnaire design, advisor, proofread, co-wrote, edited and approved the manuscript.; c Julissa Amparo Dulanto-Vargas Research Associate, Research Group in Dental Sciences, School of Dentistry, Universidad Científica del Sur, Lima, Perú. Questionnaire design, analysed the results, discussed the results, proofread and approved the manuscript.

**Keywords:** myths, oral health, pregnant women.

## Abstract

**Purpose:**

To determine oral health myths and associated factors in pregnant women.

**Materials and Methods:**

This was a cross-sectional analytical study carried out in an outpatient clinic of a public hospital in Lima, Peru, in a sample of 390 pregnant women (mean age = 30.02 ± 6.32 years) who answered a questionnaire of 61 items, comprising 39 oral health myths, 10 demographic/socioeconomic items, and 12 general health items. Multiple linear regression models were used with Jamovi v.17 at p < 0.05.

**Results:**

Oral health myths were prevalent (33.6‒77.6%) and numerous (10 [7‒13] per pregnant woman), with common gestational or maternal beliefs associated with the presence of weakening of enamel/increased risk of caries and gingivitis, infection, or calcium loss; gingival bleeding and dental caries; risks posed by spicy food, medication, radiography, or anesthesia; and intense toothbrushing. Positive predictors of oral health myths were birth in geographical districts outside Lima, previous sexually transmitted disease and pre-eclampsia. Negative predictors were having more children, a higher educational level, better employment status, minimum monthly income, and history of smoking (R^2^ = 13%; F = 2.37; p < 0.001).

**Conclusion:**

Pregnant women had a high prevalence of beliefs in a large number of oral health myths associated with birth in the geographical districts outside the capital city, less maternal experience, poorer educational, occupational and economic conditions, and obstetric-gynecological medical history.

Oral health (OH) is a crucial part of general health and should receive adequate attention throughout an individual’s life. Unfortunately, according to global indicators, OH is poor in many populations.^48^ In some groups, such as pregnant women, the OH status may worsen due to the multiple physiological and psychological changes they undergo during fetal development. The increase in certain hormones during gestation can cause tooth mobility and alter the oral bacterial environment.^34,54^

Oral disorders are largely preventable by oral hygiene practices. However, OH can be adversely impacted by socio-cultural and economic factors.^20,30,42^ A very common barrier to good individual OH is related to beliefs or myths. These are erroneous beliefs that attempt to perceive, explain or help understand a natural or social phenomenon. They are often accepted as truths despite their lack of rational explanation and professional consensus.^32^

The Health Belief Model proposed in 1950^6^ is the model most widely referred to in the different health-related sciences and is the one most frequently adapted to various branches of healthcare. It uses cognitive constructs that explain an individual’s decision-making based on the disadvantages and advantages to their health. However, an evaluation of the model found that it explained just 20% to 40% of behaviours.^6^ It has been suggested that this result neglects other aspects such as social^15^ and emotional factors.^51^

Dental sciences address oral health myths (OH-Ms) that relegate or limit attendance to dental consultations within the family only in the presence of symptoms or treatment insecurity.^2,43^ Previous studies have shown that women from different populations consider pregnancy as a risky time for dental care.^31,43^ This context could be caused by negative influencing factors, such as low educational levels, sources of information that lack scientific support, or behaviours transmitted from generation to generation.^15,31,42^

Peru is a developing country with a complex health system, and limitations in terms of access to OH care and the quality of care.^41^ Public hospitals in Peru have a protocol for the provision of dental care to pregnant women. This monitoring is important to clarify negative myths that involve maternal, fetal, and child health, as well as the health of the women themselves and those belonging to socioeconomically vulnerable groups. Therefore, the aim of this study was to evaluate the prevalence of OH-Ms and their association with demographic, socioeconomic and general health factors in pregnant women attending a Peruvian hospital.

## MATERIALS AND METHODS

### Study Design and Ethical Issues

This cross-sectional observational study was evaluated by the Institutional Research Ethics Committee of the Universidad Científica del Sur (Rº117-CIEI-CIENTÍFICA-2022) and the Institutional Research Ethics Committee of the Hospital Nacional Docente Madre Niño “San Bartolomé” (Rº734-2022-DAI-HONADOMANI-SB). The study was developed in accordance with the STROBE guideline (Table S1) and the ethical principles of the Declaration of Helsinki. The study participants provided informed consent and received OH advice and toothbrushes in appreciation of their time.

### Population and Sample

The San Bartolomé Hospital in Lima-Peru serves a population of 21,899 women aged 15 to 45 years annually in the outpatient clinic of the hospital (Source: Office of Statistics and Informatics – HONADOMANI^27^ ). This teaching hospital is considered a national reference center for specialised public care of the mother-child binomial. The study sample consisted of 390 pregnant women (mean = 30.02 ± 6.32; group < 30 years = 50%), 330 from the Obstetrics-Gynecology Department and 60 from the Odontostomatology Department.

### Selection Criteria and Sample Size

The inclusion criteria were women of Peruvian nationality who were able to answer the survey. Pregnant dental professionals and women undergoing pharmacological treatment were excluded. The minimum sample size of 318 participants was obtained with the statistical program G*Power 3.1.9.7 according to the multiple linear regression estimation of the number of pediatric OH-Ms identified in a previous pilot study (adjusted R^2^ = 0.0687), with small effect size (0.0737), confidence level (95%), power (80%) and number of predictors related to demographic, socioeconomic and general health characteristics (n = 23).

### Adaptation of the Instrument

The OH-Ms questionnaire was developed by the authors through an exhaustive literature review that included the health belief model on the behaviours that women adopt in response to the perception of susceptibility, severity, benefits, barriers, and cues to action.^6^ The questionnaire covered a number of items on OH beliefs related to the women, the pregnancy (fetus) or the baby born in populations with an economic status similar to that of Peru as a whole (low/middle).^8,24,28,33,36,46,47,48^ The OH-Ms questionnaire was evaluated by three dental experts (one radiologist and two researchers). The criteria were clarity, coherence, relevance, and sufficiency rated on a Likert scale from 1 to 4 (none, low, moderate and high). The content validity index with Aiken’s V was 1 (item and overall).

### Oral Health Myths Questionnaire 

The survey consisted of 61 items (Table S2) divided into 3 blocks: myths (39 items), demographic/socioeconomic data (10 items), and general health (12 items). The total myths were divided into: i) Individual or general myths that referred to beliefs about the OH of women as individuals, regardless of their status as pregnant or mothers (Q1−Q13); ii) gestational or pregnancy-focused myths that involved beliefs about the oral and dental health of pregnant women in relation to pregnancy, the fetus, or childbirth (Q14−Q26); iii) pediatric or infant-focused myths that involved beliefs about oral and dental health risks of mothers associated with the mother-child binomial or about their young children (Q27−Q39). The questions had three possible responses: disagree, agree, and don’t know.

### Validation and Reliability of the Questionnaire

The OH-Ms questionnaire was evaluated by three dental experts (one radiologist and two researchers). The criteria were clarity, coherence, relevance, and sufficiency rated on a Likert scale from 1 to 4 (none, low, moderate and high). The content validity index with Aiken’s V was 1 (item and overall). The questionnaire underwent a process of factorial validation and reliability to ensure comprehension within the cultural context. A pilot sample of 39 pregnant women (excluded from the study) responded with three options on a 3-point Likert scale assuming equal spacing (disagree, don’t know, agree). Exploratory factor analysis with varimax rotation in the pilot sample resulted in a Kaiser-Meyer-Olkin value of 0.500 (acceptable) and Bartlett’s test of sphericity that was statistically significant (p < 0.001), and a reliability by McDonald’s ω of 0.706 (acceptable). Factorial validation and reliability in the study sample confirmed the adequacy of the questionnaire: KMO (0.686), Bartlett’s sphericity (p < 0.001), McDonald’s ω (0.771), and factor loads (0.32 to 0.822 = moderate-strong).

### Secondary Variables and Application of the Instrument 

The demographic and socioeconomic data collected included age, place of birth, language, family type, marital status, education, employment status, economic status (minimum monthly income ≥ 1025 soles in Peru), religion, and use of social networks for health information. General health data included the number of previous children, gestational month, and medical history, e.g., urinary tract infection, sexually transmitted disease, smoking, alcohol, drugs, previous premature birth, previous low birth weight, previous miscarriage, pre-eclampsia and diabetes. The survey was administered from November 2022 to May 2023 and was conducted individually face-to-face to avoid interference with responses by technological assistance or comments from accompanying persons.

### Statistical Analysis

Statistics included frequencies, percentages, medians, interquartile range – IQR [quartile 1−3] and non-parametric Mann-Whitney U-test, Kruskal-Wallis and Spearman correlation tests (rho, ρ). Linear regression was used to analyse the main variables as continuous predictor scores based on demographic, socioeconomic and general health variables. The analysis was performed with Jamovi v.2.3.24 (The Jamovi Project) at a significance level of 0.05.

## RESULTS

Of 422 pregnant women, 25 did not agree to participate in the study and 7 did not answer the entire questionnaire (response rate = 92.42%). Table 1 presents the demographic, socioeconomic, and general health characteristics of the sample of pregnant women. The study sample of 390 pregnant women presented the following characteristics: born in the geographical district of Lima (68.2%), Spanish language (93.8%), nuclear family (47.7%), married/cohabiting (75.4%), secondary education (41%), unemployed (57.4%), average salary 1750 ± 1031 Soles (minimum monthly income = 66.4%), Catholic religion (65.1%), health information through social networks (88.7%), number of children 1 ± 1, gestational month 6 ± 2, previous urinary tract infection (34.4%), and previous miscarriage (23.1%).

**Table 1:** Demographic, socioeconomic and general health data of the study sample

Demographic/socioeconomic data
Age (years)	mean ± SD	30.02 ± 6.32
District of birth	Lima (capital of Peru)	266	68.2
	Other districts	124	31.8
Languages	Spanish	366	93.8
	Two languages*	24	6.2
Family type	Nuclear	186	47.7
Single-parent	82	21.0
Extended	68	17.4
Composite household	54	13.8
Marital status	Single	86	22.1
Married or cohabiting	294	75.4
Divorced or separated	10	2.6
Level of education	Uneducated	13	3.3
Elementary	4	1.0
High School	160	41.0
Technician	108	27.7
University student	105	26.9
Employment status	Not working / housewife only / student only	224	57.4
Occasional work	56	14.4
Part-time work	41	10.5
Full-time work	63	17.7
Household income (soles/month)	mean ± SD	1750 ± 1031
< 1025 (below minimum)	131	33.6
≥ 1025 (at or above minimum)	259	66.4
Religion	None	52	13.3
Catholic	254	65.1
Evangelical	43	11.0
Other	41	10.5
Use of social media for information	Yes	346	88.7
General health			
Number of previous children	mean ± SD	1 ± 1
None	186	47.7
One	114	29.2
Two	65	16.7
Three or more	25	6.4
Current gestational month	mean ± SD	6 ± 2	
1−3	68	17.4
4−6	92	23.6
7−9	230	59.0
Medical history	Urinary tract infection	134	34.4
Sexually transmitted disease	16	4.1
Smoking	31	7.9
Alcohol	21	5.4
Drugs	6	1.5
Previous preterm birth	24	6.2
Previous low birth weight	26	6.7
Previous abortion	90	23.1
Pre-eclampsia	30	7.7
Diabetes**	18	4.6

Table 2 shows the prevalence and number of OH-Ms among pregnant women. The prevalence and number of OH-Ms were individual myths (2.8‒36.2%; #2 [1‒3]), gestational myths (5.4‒77.6%; #4 [3‒6]), pediatric myths (2.1‒65.9%; #4 [2‒5]) and total myths (2.1‒77.6%; #10 [7‒13]). All individual myths were statistically significantly infrequent (45.4‒88.2%) (p ≤ 0.01), except for the belief about avoiding foods before tooth extraction (p = 0.670). The statistically significantly most frequent OH-Ms among pregnant women were associated with of enamel/increased risk of caries and gingivitis (77.6%), heavy toothbrushing (58.5%), spicy foods (50.8%), gingival bleeding (44.4%), and caries (42.6%). The frequency of pediatric myths statistically significantly (p < 0.05) linked infants to dental calcium loss (65.9%) and the involvement of medication (48.7%), radiography (48.2%), dental infection (40.8%) and dental anesthesia (33.6%) (p < 0.05).

**Table 2:** Myths related to oral health in pregnant women

Individual myths (average number per participant)*	2.21 ± 1.81 | 2 [1‒3]	<0.001*†
1	Only sugar causes tooth decay	254 (65.1%)a	98 (25.1%)b	38 (9.7%)c	<0.001*
2	The more you brush your teeth, the whiter they will get	267 (68.5%)a	79 (20.3%)b	44 (11.3%)c	<0.001*
3	Home recipes whiten teeth without damaging them	209 (53.6%)a	64 (16.4%)c	117 (30.0%)b	<0.001*
4	White teeth mean healthy teeth	205 (52.6%)a	141 (36.2%)b	44 (11.3%)c	0.001*
5	If you don’t have toothache, it means you have a healthy mouth	250 (64.1%)a	105 (26.9%)b	35 (9.0%)c	<0.001*
6	Avoid brushing teeth when gums bleed	255 (65.4%)a	52 (13.3%)c	83 (21.3%)b	<0.001*
7	The eruption of the wisdom tooth (third molar) increases intelligence	266 (68.2%)a	11 (2.8%)c	113 (29.0%)b	<0.001*
8	If there is no tooth pain, it is not necessary to visit the dentist	344 (88.2%)a	36 (9.2%)b	10 (2.6%)c	<0.001*
9	In-office tooth cleaning weakens teeth	307 (78.7%)a	30 (7.7%)c	53 (13.6%)b	<0.001*
10	No food should be eaten before tooth extraction	131 (33.6%)a	138 (35.4%)a	121 (31.0%)a	0.670
11	Extraction of upper teeth affects vision	177 (45.4%)a	17 (4.4%)b	196 (50.3%)a	<0.001*
12	Extraction of top teeth affects the brain	189 (48.5%)a	18 (4.6%)b	183 (46.9%)a	<0.001*
13	Extracted teeth do not need to be replaced with artificial teeth	189 (48.5%)a	72 (18.5%)c	129 (33.1%)b	<0.001*
Gestational myths (average number per participant)*	4.28 ± 2.24 | 4 [3‒6]	<0.001*†
14	Eating very cold food during pregnancy will affect the baby	229 (58.7%)a	73 (18.7%)b	88 (22.6%)b	<0.001*
15	Eating very hot food during pregnancy will affect your baby	252 (64.6%)a	35 (9.0%)c	103 (26.4%)b	<0.001*
16	Eating very spicy food during pregnancy will affect the baby	101 (25.9%)b	198 (50.8%)a	91 (23.3%)b	<0.001*
17	Pregnancy weakens teeth	57 (14.7%)b	302 (77.6%)a	30 (7.7%)c	<0.001*
18	Pregnancy causes more bleeding gums	99 (25.4%)b	173 (44.4%)a	118 (30.3%)b	<0.001*
19	Pregnancy increases tooth decay	117 (30.0%)b	166 (42.6%)a	107 (27.4%)b	0.004*
20	Pregnancy causes teeth to fall out	153 (39.2%)a	161 (41.3%)a	76 (19.5%)c	0.652
21	Do not brush your teeth during pregnancy	313 (80.3%)a	68 (17.4%)b	9 (2.3%)c	<0.001*
22	Brush your teeth more during pregnancy	114 (29.2%)b	228 (58.5%)a	48 (12.3%)c	<0.001*
23	Bleeding gums during pregnancy do not need dental care	277 (71.0%)a	76 (19.5%)b	37 (9.5%)c	<0.001*
24	If there is dental pain during pregnancy, avoid going to the dentist	284 (72.8%)a	87 (22.3%)b	19 (4.9%)c	<0.001*
25	No dental treatment during pregnancy	277 (71.0%)a	81 (20.8%)b	32 (8.2%)c	<0.001*
26	After childbirth, you should wait a few days before brushing your teeth again	329 (84.4%)a	21 (5.4%)c	40 (10.3%)b	<0.001*
Pediatric myths (average number per participant)*	3.72 ± 2.31 | 4 [2‒5]	<0.001*†
27	Oral health during pregnancy does not affect baby’s health	180 (46.2%)a	90 (23.1%)c	120 (30.8%)b	<0.001*
28	Baby extracts calcium from mother’s teeth	59 (15.1%)b	257 (65.9%)a	74 (19.0%)b	<0.001*
29	If a dental infection occurs during pregnancy, it will affect the baby	89 (22.8%)b	159 (40.8%)a	142 (36.4%)a	<0.001*
30	Dental infection does not spread between mother and baby	141 (36.2%)a	86 (22.1%)b	163 (41.8%)a	<0.001*
31	Dental infection does not spread between siblings	184 (47.2%)a	99 (25.4%)b	107 (27.4%)b	<0.001*
32	If you receive dental anesthesia during pregnancy, it will affect the baby	97 (24.9%)b	131 (33.6%)a	162 (41.5%)a	0.024*
33	If a tooth is extracted early in pregnancy, it will cause a miscarriage.	134 (34.4%)a	48 (12.3%)c	208 (53.3%)a	<0.001*
34	Taking dental drugs during pregnancy will weaken the baby	99 (25.4%)b	190 (48.7%)a	101 (25.9%)b	<0.001*
35	If you get dental x-rays during pregnancy, it will affect the baby	63 (16.2%)c	188 (48.2%)a	139 (35.6%)b	<0.001*
36	Amalgams in pregnant women are toxic to the baby	95 (24.4%)b	60 (15.4%)c	235 (60.3%)a	0.005*
37	It is not necessary to take care of children’s baby teeth as they will fall out over time	286 (73.3%)a	78 (20.0%)b	26 (6.7%)c	<0.001*
38	Children’s exfoliated teeth kept in a special place bring good luck	198 (50.8%)a	58 (14.9%)c	134 (34.4%)b	<0.001*
39	If a baby is born with teeth, it brings bad luck	250 (64.1%)a	8 (2.1%)c	132 (33.8%)b	<0.001*
Total myths (average number per participant)*	10.21 ± 4.84 | 10 [7‒13]	<0.001*†

Table 3 shows the number of OH-Ms according to the characteristics of the pregnant women. A lower number of OH-Ms per pregnant woman was statistically significantly associated with higher education (individual myths = 2, gestational myths = 4), working full time (individual myths = 1, gestational myths = 4, pediatrics myths = 3, total myths = 9), higher economic level (ρ individual myths = −0.158; gestational myths = −0.108), not using social networks for health information (gestational myths = 4, total myths = 8), higher gestational month (ρ individual myths = −0.101; pediatric myths = −0.134; total myths = −0.135), no history of sexually transmitted disease (pediatric myths = 3, total myths = 10), smoking (gestational myths = 4), preterm birth (pediatric myths = 3), or pre-eclampsia (individual myths = 2, total myths = 10) (p < 0.05).

**Table 3:** Comparisons of number of oral health myths according to the characteristics of the pregnant women

Demographics/socioeconomic data
Age	Years^†^	0.139	0.832	0.700	0.558
Age group	Intergroup^‡^	0.093	0.957	0.881	0.750
District of birth	Intergroup^¥^	0.448	0.486	0.692	0.720
Languages	Intergroup^¥^	0.119	0.589	0.140	0.072
Family type	Intergroup^¥^	0.779	0.588	0.371	0.292
Marital status	Intergroup^¥^	0.761	0.131	0.155	0.184
Level of education	Intergroup^¥^	<0.001*	0.031*	0.219	0.067
Uneducated	Me [Q1−Q3]	3 [2−4]ab	4 [3−5]a		
Elementary	Me [Q1−Q3]	4.5 [3.5−7]a	5 [2−8]a		
High School	Me [Q1−Q3]	2 [1−3]b	5 [3−6]a		
Technician	Me [Q1−Q3]	2 [1−3]b	4 [3−5]a		
Superior	Me [Q1−Q3]	2 [1−2]b	4 [2−5]b		
Employment status	Intergroup^¥^	0.015*	0.028*	0.019*	0.017*
Not working, only housewife/student	Me [Q1−Q3]	2 [1−3]a	4 [3−6]a	4 [2−6]a	10 [8−13]a
Occasional work	Me [Q1−Q3]	2 [1−3]ab	4 [2.5−5]ab	3 [1−4]b	9 [5−13]ab
Part-time work	Me [Q1−Q3]	2 [1−3]ab	4 [3−6]a	4 [2−6]a	10 [8−13]ab
Full-time work	Me [Q1−Q3]	1 [1−2]b	4 [2−5]b	3 [2−5]b	9 [5−12]b
Household income	Soles^†^	0.002*	0.033*	0.900	0,055
	ρ	−0.158	−0.108		
Minimum income (soles/month)	Intergroup^¥^	<0.001*	0.104	0.872	0.030*
<1025	Me [Q1−Q3]	2 [1−4]a			11 [7−13]a
≥1025	Me [Q1−Q3]	2 [1−3]b			10 [6−13]b
Religion	Intergroup^¥^	0.549	0.576	0.738	0.602
Use of social media for health	Intergroup^‡^	0.088	0.032*	0.169	0.048*
No	Me [Q1−Q3]		4 [2−5]		8 [5−14]
Yes	Me [Q1−Q3]		4 [3−6]		10 [7−13]
General health					
Previous children	Number^†^	0.693	0.116	0.456	0.221
Current gestational month	Months^†^	0.046*	0.286	0.008*	0.007*
	ρ	−0.101		−0.134	−0.135
Medical history					
Urinary tract infection	Intergroup^‡^	0.620	0.223	0.051	0.271
Sexually transmitted disease	Intergroup^‡^	0.480	0.071	0.009*	0.017*
No	Me [Q1−Q3]			3 [2−5]	10 [7−13]
Yes	Me [Q1−Q3]			6 [4−6]	14 [10−16]
Smoking	Intergroup^‡^	0.685	0.004*	0.597	0.256
No	Me [Q1−Q3]		4 [3−6]		
Yes	Me [Q1−Q3]		5 [4−7]		
Alcohol	Intergroup^‡^	0.611	0.810	0.057	0.530
Drugs	Intergroup^‡^	0.830	0.990	0.170	0.588
Previous preterm birth	Intergroup^‡^	0.359	0.651	0.044*	0.172
No	Me [Q1−Q3]			3 [2−5]	
Yes	Me [Q1−Q3]			5 [3−6]	
Previous low birth weight	Intergroup^‡^	0.281	0.593	0.160	0.329
Previous abortion	Intergroup^‡^	0.893	0.430	0.959	0.714
Pre−eclampsia	Intergroup^‡^	0.002*	0.132	0.353	0.039*
No	Me [Q1−Q3]	2 [1−3]			10 [7−13]
Yes	Me [Q1−Q3]	3 [2−5]			13 [9−14]
Diabetes	Intergroup^‡^	0.856	0.758	0.664	0.803

Table 4 presents the multiple linear regression model of the number of OH-Ms according to the characteristics of the pregnant women. Multiple regression models showed statistical significance for individual myths (R^2^ = 17.9%; F = 3.47; p < 0.001), gestational myths (R^2^ = 13.1%; F = 2.38; p < 0.001), and total myths (R^2^ = 13%; F = 2.37; p < 0.001) but not for pediatric myths (p > 0.05). Predictors of individual myths were positive in women born in geographical districts outside Lima (β = 0.481; p = 0.016) and women with previous pre-eclampsia (β = 1.46; p < 0.001), and negative in women with a higher educational level (β = −0.343; p < 0.001) and with the minimum monthly income (β = −0.709; p = 0.002). Gestational myths were positive for previous pre-eclampsia (β = 1.153; p = 0.008) and negative for better employment status (β = −0.228; p = 0.027), more children (β = −0.413; p = 0.003), and smoking (β = −1.86; p < 0.001). Predictors in total myths were positive for the history of sexually transmitted disease (β = 2.625; p = 0.039) and pre-eclampsia (β = 3.36; p < 0.001), and negative for more children (β = −0.599; p = 0.041) and smoking (β = −2.602; p = 0.023).

**Table 4:** Multiple linear regression analysis of the number of oral health myths according to the characteristics of the pregnant women

Interception	4.456	0.666	<0,001*	4.551	0.849	<0.001*	3.328	0.899	<0.001*	12.335	1.829	<0.001*
Age	−0.024	0.017	0.154	0.029	0.021	0.178	−0,.010	0.023	0.644	−0.006	0.046	0.904
District (Lima)	0.481	0.199	0.016*	0.210	0.254	0.409	0.282	0.269	0.295	0.973	0.547	0.076
Language (Spanish)	−0.215	0.371	0.562	0.080	0.473	0.866	−0.119	0.501	0.812	−0.255	1.019	0.803
Type of family (nuclear or extended, with or without children)	−0.099	0.189	0.601	−0.018	0.241	0.942	0.185	0.255	0.468	0.069	0.519	0.895
Marital status (single, divorced, separated)	0.111	0.224	0.621	−0.417	0.285	0.145	−0.230	0.302	0.447	−0.536	0.615	0.384
Level of education	−0.343	0.098	<0.001*	−0.200	0.124	0.108	0.151	0.132	0.253	−0.392	0.268	0.144
Employment status	−0.109	0.080	0.175	−0.228	0.103	0.027*	−0.045	0.109	0.677	−0.383	0.221	0.084
Household income	1.194	1.094	0.273	−1.814	1.384	0.192	2.074	1.474	0.159	1.454	2.984	0.627
Minimum living income (no)	−0.709	0.230	0.002*	0.044	0.293	0.882	−0.288	0.310	0.353	−0.954	0.631	0.131
Religion (non−Catholic)	0.095	0.188	0.614	−0.255	0.240	0.288	−0.110	0.254	0.666	−0.270	0.516	0.601
Use of social media for health (no)	0.320	0.282	0.257	0.631	0.360	0.080	0.434	0.381	0.256	1.385	0.775	0.075
Number of previous children	−0.168	0.106	0.114	−0.413	0.136	0.003*	−0.019	0.144	0.897	−0.599	0.292	0.041*
Current gestational month	−0.056	0.039	0.150	0.019	0.050	0.700	−0.087	0.053	0.099	−0.124	0.107	0.248
Urinary tract infection (no)	−0.123	0.190	0.517	0.253	0.242	0.295	0.431	0.256	0.093	0.561	0.521	0.282
Sexually transmitted disease (no)	0.306	0.461	0.508	0.946	0.588	0.108	1.373	0.622	0,.28*	2.625	1.266	0.039*
Smoking (no)	0.140	0.414	0.736	−1.860	0.528	<0.001*	−0.882	0.559	0.115	−2.602	1.136	0.023*
Alcohol (no)	−0.241	0.531	0.650	1.172	0.677	0.084	1.230	0.717	0.087	2.161	1.458	0.139
Drugs (no)	−0.411	0.817	0.615	−0.475	1.042	0.649	0.329	1.103	0.766	−0.557	2.244	0.804
Previous preterm birth (no)	−0.315	0.427	0.461	0.240	0.545	0.660	1.019	0.577	0.078	0.943	1.173	0.422
Previous low birth weight (no)	0.687	0.417	0.101	0.257	0.532	0.629	0.012	0.564	0.983	0.956	1.146	0.405
Previous abortion (no)	−0.076	0.233	0.745	0.161	0.297	0.589	−0.371	0.315	0.240	−0285	0.641	0.656
Pre-eclampsia (no)	1.460	0.340	<0.001*	1.153	0.434	0.008*	0.747	0.459	0.104	3.360	0.934	<0.001*
Diabetes (no)	−0.115	0.434	0.792	−0.255	0.553	0.645	−0.249	0.586	0.671	−0.618	1.191	0.604

## DISCUSSION

The literature has shown that some pregnant women have positive OH practices and attitudes, such as brushing their teeth and attending dentist appointments.^3^ On the other hand, some women may hold culturally transmitted misbeliefs that may affect their OH behaviours, making them susceptible to uncertainty as to whether something will harm their child.^42^ This study found that the prevalence of OH-Ms among pregnant Peruvian women was 33.6% to 77.6%m with 7 to 13 myths per person, and that these were associated with their place of birth, educational level, economic status, and a history of pre-eclampsia, sexually transmitted disease or previous children.

This majority of the participants (48.5% to 88.2%) did not agree with 10 of the 13 individual OH-Ms evaluated, similar to what was described in studies performed in Brazil,^13^ India,^45,47,49,50,52^ Pakistan,^32,33^ Saudi Arabia,^10,24,25,36,46^ and the United States.^16,53^ These OH-Ms were as follows: a visit to the dentist is made only when there are symptoms,^16,24,25,32,36,50,53^ professional tooth cleaning in the dental office weakens the teeth,^24,32,46,47,49,50,53^ intense toothbrushing whitens the teeth,^24,46^ the eruption of the third molar increases intelligence,^24,47^ toothbrushing is avoided when gingiva bleed,^45,46,52^ only sugar causes tooth decay,^13,46^ white teeth^50^ or painless teeth indicate OH,^33^ home tooth whiteners do not damage teeth^10,47^ and missing teeth do not need to be prosthetically replaced.^24,25^ However, there were also discrepancies with populations from North and South India, who associated the need for dental visits with dental pain^52^ and sensitivity, and that tooth mobility is caused by dental cleaning.^49,52^ These findings are useful for the design of preventive promotional programs in OH which take into account the characteristics of disadvantaged demographic groups.^2,20,30,31^

Regarding other individual OH-Ms, a statistically significant number of the study participants were skeptical about the association of tooth extractions with eye and brain injuries, which was consistent with participants from India^37,49,52^ and Pakistan.^33^ In contrast, the participants in studies in Indonesia,^40^ Saudi Arabia^24^ and southern India^47^ did not agree with these myths. These beliefs could be influenced by the higher perception of risk given the proximity of oral surgery to delicate structures of the orbital cavity. Participants could also have received information about negative experiences arising from vision complications due to the use of local anesthesia.^37,49^ Regarding the negative myth of avoiding food before tooth extraction, in this study there were divided opinions, as also shown in Saudi Arabia.^24^ It is possible that this myth is associated with preoperative fasting before sedation or anesthesia commonly indicated in major surgical interventions. Therefore, this belief can be detrimental to the recovery of the dental patient due to post-surgery food restriction.^5^

This study found that 58.7% to 84.4% of the pregnant women participating in the study disagreed with 7 of the 13 gestational OH-Ms analysed, similar to beliefs among pregnant women in US,^16^ Italian,^3^ Indian,^28,39,45,47^ Indonesian,^40^ Nepalese,^11^ Nigerian,^1^ Northern Peruvian^8^ and Saudi^46^ studies. These myths included the need for pregnant women to avoid brushing for a few days during^28^ or after delivery,^40,47^ dental visits for dental pain,^1,3,11,28^ avoidance of dental treatments,^8,16,28,39,40,45,46^ the idea that gingival bleeding was normal during pregnancy^28,40^ and can be affected by cold/hot foods.^28,40^ In contrast, multiple pregnant populations accepted the myth of avoiding dental treatments as they were considered unsafe.^18,22,24,26,29,42,44,52^ The positive results of this study promote good oral hygiene practices, visits to the dentist^4,22,30^ and a healthy diet among pregnant women.^28,36^ It is necessary to counter the inaccuracies of information generated by family advice^17,28,43^ and unreliable internet content,^2,38,44,46^ especially when pregnant women should be motivated and have a positive attitude towards OH care.^23^

In relation to other OH-Ms during pregnancy, this study found that, similar to pregnant women in US,^23^ Brazil,^14^ Indian,^26,28,39^ Italian,^3^ Nigerian,^1^ Saudi,^9,36^ and Turkish^17^ studies, there was frequent agreement (42.6% to 77.6%) that pregnancy was associated with of enamel/increased risk of caries and gingivitis,^9,14,26^ more toothbrushing,^28^ the idea that spicy foods should be avoided,^36^ increased gingival bleeding^3,9,17,23,26,39^ and caries.^1,9^ This was in contrast to studies from multiple regions that disagreed with the involvement of pregnancy in gingival bleeding^1,8,45^ and caries.^8,23^ Regarding the myth of pregnancy as a factor causing tooth loss, the responses of disagreement and agreement were similarly distributed. This belief was the most accepted according to a systematic review,^29^ in contrast to other studies reporting disagreement with this myth.^1,7,8,11,12,14,23^ The lack of an interdisciplinary approach to providing OH care as part of the monitoring of pregnant women may be a barrier to eliminating many OH-Ms held by pregnant women.^2,17,23,38,39,42,44^ Another obstacle may be that some some dentists hesitate to provide dental care to the pregnant, due to the risk of possible legal claims that pregnant women may make, should they associate some congenital defect or a spontaneous pre-term birth with dental treatment.^5,23,42,43^

This study showed that, similar to the results described in US,^12,23^ Brazilian,^13,14^ Croatian,^21^ Indian,^28,47,50,52^ Malaysian,^44^ Northern Peruvian^8^ and Saudi^24,36,46^ populations, 36.2% to 73.3% of the participants significantly disagreed with 5 of the 13 pediatric OH-Ms related to deficiencies in the care of deciduous teeth,^13,14,36,46,50^ a connection between bad luck/good luck and natal teeth^24,52^ or exfoliated teeth,^24^ respectively, dental infection transmitted between siblings^24,47,52^ or from the mother,^8,23^ and not relating gestational OH to that of the child.^12,13,14,21,28,44,52^ In other studies of pregnant women, however, the participants agreed with myths about the negligible importance of deciduous dentition^24,28,32,33,52^ or associating deciduous-tooth loss with luck,^52^ while not considering the relationship between the health of the child and maternal OH.^4,26,35,38^ The pediatric myths rejected in this study promote the performance of dental visits during pregnancy^17,40^ to reduce the risk of oral diseases.^19,34^

Pregnant women in this study frequently agreed (33.6% to 65.9%) with five myths about the effect of the baby on maternal dental calcium,^7,8,13,17,38,46^ the risk to the child during pregnancy due to dental infection,^7,12,17^ dental medications,^17,23,29,38,40,52^ dental x-rays^17,23,29,35^ and dental anesthesia,^14,17,23,35,38,40^ similar to what has been described in US,^12,23^ Brazilian,^13,14^ Canadian,^7^ Indian,^29,52^ Indonesian,^40^ Northern Peruvian,^8^ Spanish,^35^ Saudi^46^ and Turkish^17,38^ populations. However, some pregnant women from various regions did not think that the baby can take calcium from the mother‘s teeth,^4,12^ or that medications,^8,14,45^ x-rays,^8,9,14,45^ and anesthesia^8,9,21,28^ could harm the fetus. Among the remaining pediatric myths, pregnant women doubted whether the mother’s amalgams affect the child and that extraction induces miscarriage. This last myth was rejected by other studies.^17,28,40^ The acceptance of the myths of the pregnant women in this study could be due to the fear of negatively affecting fetal development^42,43,51^ or by the caution indicated in the first trimester of pregnancy due to the risk of compression of the inferior vena cava and discomfort due to the gag reflex.^5,21,29,42^

As described by other authors, this study found that better educational^8,18,24,28,33,35,37,40,42,46^ labor,^35,42^ and economic conditions,^2,18,28,36,42^ as well as previous maternal experience,^4,28^ were negative predictors of OH-Ms. Interrelated factors, such as improving the availability and selection of quality information on OH, can can increase health literacy.^14,31,43^ Furthermore, in addition to OH advice received in previous prenatal check-ups,^3,14^ OH-Ms can be counteracted by better access to professional care and information, with shorter waiting times at the dental practice and patient-friendly working hours, along with paid or co-paid dental services.^4,7,15,22,30,42,45^ Agreement with other studies existed in relation to beliefs rooted in geographic location^3,10,28,29,33,35,42,49^ or adverse gestational history, such as pre-eclampsia and sexually transmitted diseases,^3,23,28,42^ which were identified as positive predictors of myths. In light of these associations, it is essential to focus on mothers as an important source of information for the family,^36,40^ making it necessary to provide OH programs with culturally sensitive care strategies^2,22^ that generate reasoned actions for behavioural changes.^33^ It is also important for dentists to help raise awareness about oral care based on the determinants that predict erroneous beliefs.^15,23,42^

The population of this study included pregnant Peruvian women receiving obstetric-gynecological care in Lima, but it should be taken into account that these results may differ in pregnant women seeking care in cities other than the capital of Peru. The study questionnaire has a subjective nature that could affect the accuracy of the information. Moreover, closed questions were used that could have restricted the discovery of other health beliefs. The multivariate design allowed determination of the factors associated with OH-Ms, but the cross-sectional design limits determining causality among the variables.

## CONCLUSION

Within these limitations, the prevalence and number of oral health myths in pregnant Peruvian women were high and were associated with being born outside the capital, having less maternal experience, a low educational level, low occupational and economic status, and their own obstetric-gynecological medical history.

**Table S1:** STROBE Statement‒Checklist of items that should be included in reports of cross-sectional studies

Title and abstract	1	(a) Indicate the study’s design with a commonly used term in the title or the abstract	1
(b) Provide in the abstract an informative and balanced summary of what was done and what was found	1
Introduction
Background/rationale	2	Explain the scientific background and rationale for the investigation being reported	2–3
Objectives	3	State specific objectives, including any prespecified hypotheses	3
Methods
Study design	4	Present key elements of study design early in the paper	3
Setting	5	Describe the setting, locations, and relevant dates, including periods of recruitment, exposure, follow-up, and data collection	4
Participants	6	(a) Give the eligibility criteria, and the sources and methods of selection of participants	4
Variables	7	Clearly define all outcomes, exposures, predictors, potential confounders, and effect modifiers. Give diagnostic criteria, if applicable	5–6
Data sources/ measurement	8*	For each variable of interest, give sources of data and details of methods of assessment (measurement). Describe comparability of assessment methods if there is more than one group	NA
Bias	9	Describe any efforts to address potential sources of bias	6
Study size	10	Explain how the study size was arrived at	4
Quantitative variables	11	Explain how quantitative variables were handled in the analyses. If applicable, describe which groupings were chosen and why	5
Statistical methods	12	(a) Describe all statistical methods, including those used to control for confounding	6
(b) Describe any methods used to examine subgroups and interactions	6
(c) Explain how missing data were addressed	6
(d) If applicable, describe analytical methods taking account of sampling strategy	6
(e) Describe any sensitivity analyses	6
Results
Participants	13*	(a) Report numbers of individuals at each stage of study—eg numbers potentially eligible, examined for eligibility, confirmed eligible, included in the study, completing follow-up, and analysed	7
(b) Give reasons for non-participation at each stage	7
(c) Consider use of a flow diagram	NA
Descriptive data	14*	(a) Give characteristics of study participants (eg demographic, clinical, social) and information on exposures and potential confounders	7 Table 1
(b) Indicate number of participants with missing data for each variable of interest	7
Outcome data	15*	Report numbers of outcome events or summary measures	7–8 Table 2
Main results	16	(a) Give unadjusted estimates and, if applicable, confounder-adjusted estimates and their precision (eg, 95% confidence interval). Make clear which confounders were adjusted for and why they were included	7–8 Table 3 and 4
(b) Report category boundaries when continuous variables were categorized	NA
(c) If relevant, consider translating estimates of relative risk into absolute risk for a meaningful time period	NA
Other analyses	17	Report other analyses done—eg analyses of subgroups and interactions, and sensitivity analyses	NA
Discussion
Key results	18	Summarise key results with reference to study objectives	8–9
Limitations	19	Discuss limitations of the study, taking into account sources of potential bias or imprecision. Discuss both direction and magnitude of any potential bias	13
Interpretation	20	Give a cautious overall interpretation of results considering objectives, limitations, multiplicity of analyses, results from similar studies, and other relevant evidence	9–12
Generalisability	21	Discuss the generalisability (external validity) of the study results	13
Other information
Funding	22	Give the source of funding and the role of the funders for the present study and, if applicable, for the original study on which the present article is based	Title page

**Table S2a:** Survey used in this study (English version)

Q1	Only sugar causes tooth decay			
Q2	The more you brush your teeth, the whiter they will become			
Q3	Homemade recipes whiten teeth without damaging them (e.g., salt, vinegar, lemon or baking soda)			
Q4	White teeth mean healthy teeth			
Q5	If you don’t have toothache, it means you have a healthy mouth			
Q6	Avoid brushing teeth when gums bleed			
Q7	The eruption of the wisdom tooth (third molar) increases intelligence			
Q8	If there is no tooth pain, it is not necessary to visit the dentist			
Q9	In-office tooth cleaning weakens teeth			
Q10	No food should be eaten before tooth extraction.			
Q11	Extraction of upper teeth affects vision			
Q12	Extraction of top teeth affects the brain			
Q13	Extracted teeth do not need to be replaced with artificial teeth			
Q14	Eating very cold food during pregnancy will affect the baby			
Q15	Eating very hot food during pregnancy will affect your baby			
Q16	Eating very spicy food during pregnancy will affect the baby			
Q17	Pregnancy weakens teeth			
Q18	Pregnancy causes more bleeding gums			
Q19	Pregnancy increases tooth decay			
Q20	Pregnancy causes teeth to fall out			
Q21	Do not brush your teeth during pregnancy			
Q22	Brush your teeth more during pregnancy			
Q23	Bleeding gums during pregnancy do not need dental care			
Q24	If there is dental pain during pregnancy, avoid going to the dentist			
Q25	No dental treatment during pregnancy			
Q26	After childbirth, you should wait a few days before brushing your teeth again			
Q27	Oral health during pregnancy does not affect baby’s health			
Q28	Baby extracts calcium from mother’s teeth			
Q29	If a dental infection occurs during pregnancy, it will affect the baby			
Q30	Dental infection does not spread between mother and baby			
Q31	Dental infection does not spread between siblings			
Q32	If you receive dental anesthesia during pregnancy, it will affect the baby			
Q33	If a tooth is extracted early in pregnancy, it will cause a miscarriage			
Q34	Taking dental drugs during pregnancy will weaken the baby			
Q35	If you get dental x-rays during pregnancy, it will affect the baby			
Q36	Amalgams in pregnant women are toxic to the baby			
Q37	It is not necessary to take care of children’s baby teeth as they will fall out over time			
Q38	Children’s exfoliated teeth kept in a special place bring good luck			
Q39	If a baby is born with teeth, it brings bad luck			

**Table S2b:** Survey used in this study (Spanish version)

N°	Preguntas sobre mitos de salud oral individuales, gestacionales y pediátricos en gestantes	Opciones de respuestas
En desacuerdo	De acuerdo	No sé
P1	Solo el azúcar causa caries en los dientes			
P2	Cuando más te cepillas los dientes, más blancos serán			
P3	Las recetas caseras blanquean los dientes sin dañarlos (Ej. sal, vinagre, limón o bicarbonato de sodio)			
P4	Los dientes blancos significan dientes sanos			
P5	Si no tienes dolor de dientes significa que tienes una boca sana			
P6	Se debe evitar lavarse los dientes cuando las encías sangran			
P7	La erupción del diente de juicio (tercer molar) aumenta la inteligencia			
P8	Si no hay dolor de diente, no es necesario visitar al dentista			
P9	La limpieza dental en el consultorio debilita los dientes			
P10	No se debe comer alimentos antes de la extracción del diente			
P11	La extracción de los dientes de arriba afecta la visión ocular			
P12	La extracción de los dientes de arriba afecta el cerebro			
P13	Los dientes extraídos no necesitan reemplazarse con dientes artificiales			
P14	Comer alimentos muy fríos durante el embarazo afectará al bebé			
P15	Comer alimentos muy calientes durante el embarazo afectará al bebé			
P16	Comer alimentos muy picantes durante el embarazo afectará al bebé			
P17	Los dientes se debilitan a causa del embarazo			
P18	El embarazo provoca que sangren más las encías			
P19	El embarazo aumenta las caries en los dientes			
P20	El embarazo provoca que se caigan los dientes			
P21	No se debe cepillar los dientes durante el embarazo			
P22	Se debe cepillar más los dientes durante el embarazo			
P23	El sangrado de las encías durante el embarazo no necesita atención dental			
P24	Si hay dolor dental durante el embarazo, se debe evitar acudir al dentista			
P25	No se debe recibir tratamiento dental durante el embarazo			
P26	Después del parto, se debe esperar unos días para volver a cepillarse los dientes			
P27	La salud oral durante el embarazo no afecta a la salud del bebé			
P28	Los bebés extraen calcio de los dientes de la madre			
P29	Si se presenta una infección dental durante el embarazo, afectará al bebé			
P30	La infección dental no se contagia entre la madre y el bebé			
P31	La infección dental no se contagia entre hermanos			
P32	Si se recibe anestesia dental durante el embarazo, afectará al bebé			
P33	Si se extrae un diente al inicio del embarazo, provocará un aborto espontáneo			
P34	El consumo de medicamentos dentales durante el embarazo debilitará al bebé			
P35	Si se recibe radiografía dental durante el embarazo, afectará al bebé			
P36	Las amalgamas que tengan las embarazadas son tóxicas para el bebé			
P37	No es necesario cuidar los dientes de leche de los niños ya que se caerán con el tiempo			
P38	Los dientes caídos de los niños guardados en un lugar especial traen buena suerte			
P39	Si un bebé nace con dientes, trae mala suerte			
				
